# Can Sense of Opportunity Identification Efficacy Play a Mediating Role? Relationship Between Network Embeddedness and Social Entrepreneurial Intention of University Students

**DOI:** 10.3389/fpsyg.2019.01342

**Published:** 2019-06-11

**Authors:** Wenke Wang, Yingkai Tang, Yao Liu, Tao Zheng, Jing Liu, Haiyue Liu

**Affiliations:** ^1^Business School, Sichuan Normal University, Chengdu, China; ^2^Business School, Sichuan University, Chengdu, China; ^3^Scientific Research Department, Sichuan Normal University, Chengdu, China

**Keywords:** network embeddedness, sense of opportunity identification efficacy, social entrepreneurial intention, university students, mediating role

## Abstract

Social entrepreneurship is an entrepreneurial activity centering on solving social problems and creating social values, which can effectively alleviate the problems of sustainable development such as an excessive gap between the rich and the poor, a lack of resources and so on, and can resolve the “triple failures” of government, the market and public welfare departments to a certain extent. As the subjective attitude of entrepreneurs, entrepreneurial intention can predict a representational incidence rate of entrepreneurial behavior. Therefore, aimed at university students, a special group of entrepreneurs, this paper constructs a theoretical framework of “network embeddedness – sense of opportunity identification efficacy – university students’ social entrepreneurial intention” from the perspective of social cognitive theory through 466 pieces of valid survey data, and uses Stata 14 to construct a multiple linear regression model to explore the mechanism of action between the three. The results show that university students’ sense of opportunity identification efficacy can significantly and positively stimulate their social entrepreneurial intention, and the network embeddedness (network scale and network intensity) of entrepreneurs is also significantly and positively correlated with their sense of opportunity identification efficacy; however, via the hierarchical regression model, it was found that the sense of opportunity identification efficacy can only partially mediate the relationship between network embeddedness and university students’ social entrepreneurial intention, which is mainly manifested in the positive correlation between university students’ social entrepreneurial intention and their network scale, and is unrelated to network intensity. This research contributes to enriching the theory of social entrepreneurial intention and guides the strengthening of university students’ social entrepreneurial intention in reality.

## Introduction

The continuing expansion of enrollment in universities sees a progressive increase of university graduates every year; however, the continuing downward trend of economic growth has added increasing pressure on graduates in their search for employment. Therefore, entrepreneurship has become a popular method for university graduates when facing fierce social competition and the considerable pressure to find employment. This paper will focus primarily on one of the paths university students take in entrepreneurial behavior: the subdivision of social entrepreneurship.

Social entrepreneurship is a new entrepreneurship model (Mair and Marti, 2006) primarily designed to realize social value, but also with the characteristics of commercial enterprises. Its source comes from the “triple failures” of the government, the market and the public welfare department. It is a social opportunity that can satisfy social entrepreneurs that are not satisfied by commercial enterprises or through traditional government and public welfare means (Jin and Liu, 2015). It can play a greater role in alleviating modern social problems such as resource shortages and environmental pollution (Austin et al., 2012). Therefore, intensifying the university students’ entrepreneurial strength of social enterprises is helpful in accelerating social progress and building a harmonious society, which has very important practical significance (Peredo and Mclean, 2006; Xue, 2016). However, entrepreneurial intention is considered as the best predictor of entrepreneurs’ potential entrepreneurial behavior, and is a subjective attitude expression of entrepreneurs’ willingness to engage in entrepreneurial activities (Krueger and Carsrud, 1993; Mu, 2007), therefore the prediction and promotion of the transformation rate of entrepreneurial behavior can be realized through research on university students’ social entrepreneurial intention.

At present, scholarly research on social entrepreneurial intention mainly focuses on meaning (Anderson et al., 2010; Morris et al., 2011), measure (Kraus et al., 2012; Dwivedi and Weerawardena, 2018), the individual characteristics and entrepreneurial background of entrepreneurs, and the impact of social capital on entrepreneurial intention (Fan and Wang, 2005), the impact of opportunity identification on entrepreneurial intention and the role of entrepreneurs in opportunity identification (Zahra et al., 2009; Corner and Ho, 2010; Conger et al., 2012; Alvarez et al., 2014), the relationship between entrepreneurial motivation and entrepreneurial intention (Monteiro et al., 2013; Hockerts, 2015), the role of the national system on entrepreneurial intention (Estrin et al., 2013; Mendoza et al., 2015), etc. Moreover, Liu and Zhuang (2018) found that due to the lack of systematic theoretical construction, existing research cannot further reveal the deep logical relationship between the motivation, process and performance of social entrepreneurship through combing the quantitative research documents on social entrepreneurship published in international mainstream management journals since 2005, and they suggested that the internal mechanism of social entrepreneurship should be deeply explored through intermediary variables. Generally speaking, most of the current research on social entrepreneurial intention focuses on the individual characteristics of entrepreneurs, while research into the influence of the external environment on entrepreneurial intention is very scarce.

However, it is well known that the external environment in which entrepreneurs live, that is, their network embeddedness, has a direct impact on their decision-making and behavior in the entrepreneurial process (Zadek et al., 1997; Zahra et al., 2009). Network embeddedness is a specific relationship structure between people and their social network, the source for entrepreneurs to obtain capital and support, and key to promoting the alliance between enterprises and economic relations (Wenman, 2018). Therefore, different resources and information represented by the diverse social network embeddedness of entrepreneurs will affect the intensity of their social entrepreneurial intention. However, in recent years, many scholars have gradually added the individual psychological factors of entrepreneurs to research on social networks and their entrepreneurial behavior. For example, Bandura (2000) believes that the network embeddedness of entrepreneurs is different, and their difficulty in obtaining entrepreneurial information and resources is also distinct, thus affecting the four major senses of efficacy, namely innovation, opportunity identification, risk tolerance and relationship coordination, and finally showing the diversified entrepreneurial intentions and behaviors of entrepreneurs. However, this paper holds that the identification and mastery of various opportunities in the entrepreneurial process can directly determine the development status and survival probability of the enterprise, and good opportunity identification ability gives the possibility for entrepreneurs to implement actions, achieve goals and establish new enterprises (Hills et al., 2011). Xie (2017) believes that opportunity identification in the entrepreneurial process is the personal summary of cognitive processes and entrepreneurial activities, and is the precursor of various entrepreneurial actions such as entrepreneurs’ evaluation and exploration of entrepreneurial opportunities. Therefore, in the current dynamic environment with information network asymmetry and high uncertainty, attention to the opportunity identification efficacy induction of entrepreneurs is one of the key issues in the field of entrepreneurship research (Shane and Venkataraman, 2001).

However, current research does not make precise divisions of social entrepreneurs’ backgrounds and research into the social entrepreneurial intention of university students is almost non-existent, despite the fact that many people regard university students one of the groups with the greatest potential for innovation and entrepreneurship as a main force in China’s entrepreneurial landscape. Under the guidance of the strategy to become an innovation-oriented country, the state of social entrepreneurship education in Chinese universities has been continuously optimized. However, there still exist problems such as cognitive deviation of educational concepts, a disjointed curriculum system, and the incomplete state of social entrepreneurship education support and guarantee systems (Qiu, 2018). That are well behind developed nations such as the United States and Japan. Therefore deviations in educational concepts, curriculum systems, guarantee systems and so on, impair university students’ social entrepreneurial intention to a certain degree (Liu, 2018). In this educational environment, discovering how to research the voluntary social entrepreneurial intention of students from their own point of view and their social relations structure and network embeddedness and improve university students’ social entrepreneurial intention at the source to realize a conversion rate of their social entrepreneurial behavior is of relatively important research urgency and practical significance.

To summarize, this paper selects the network embeddedness of Chinese university students as its independent variable, and the social entrepreneurial intention of university students as its dependent variable, and introduces the sense of opportunity identification efficacy as its intermediary variable, and constructs the theoretical model of “network embeddedness-sense of opportunity identification efficacy-social entrepreneurial intention of university students,” and discusses the action mechanism and influence mechanism between network embeddedness, sense of opportunity identification efficacy and the social entrepreneurial intention of university students in China. The possible contributions of this paper lie in the following aspects: (1) Enrich theoretical research on network embeddedness and social entrepreneurship, particularly social entrepreneurial intention, and apply survey data as a support to empirically explore the logical relationship between the two; (2) From the perspective of psychological cognition, apply university students’ own sense of opportunity identification efficacy to guide and deepen their entrepreneurial intention so as to enrich theoretical research on the sense of opportunity identification efficacy; (3) Use the results of this paper to improve the conversion rate of Chinese university students’ social entrepreneurship, promote the achievement and realization of university students’ social mission, and improve China’s social entrepreneurship rate, to compensate for the deficiencies of government failure, market failure and charity failure to a certain extent, and contribute more to China’s poverty eradication, employment solution and social environment improvement.

## Literature Review and Hypothesis Development

### Social Entrepreneurial Intention and Its Theory

Krueger and Carsrud (1993) believe that entrepreneurial intention is the best indicator to predict entrepreneurial behavior. Because people’s behavior is an intentional decision made through pre-judgment of their own environment, entrepreneurial behavior originates from the emergence of entrepreneurial intention. Dwivedi and Weerawardena (2018) define social entrepreneurial intention as an attitude composed of innovation, initiative, risk management, effect orientation, social mission orientation and sustainable orientation. Its purpose is to solve social market failure and create greater social value.

At present, the most widely used and credible theories on social entrepreneurial intention are the Theory of Planned Behavior and the Revised Model of Entrepreneurial Intention. The Theory of Planned Behavior was put forward by Professor Ajzen (1991), who believes that people can control their behavior themselves and make timely adjustments according to changes in their environment. Of the five main factors emphasized in this theory, attitude, subjective norm, perceived behavioral control, behavior intention and behavior, Ajzen (1991) believes that there is a correlative relationship between entrepreneurial intention and the positive nature, normalization and degree of behavioral control of an entrepreneur’s attitude. The more positive the behavioral subject is, the more support from significant others they receive, and the better perceived behavioral control they have, the more behavioral intention they get; contrarily becoming smaller. Of these, correct perceived behavioral control reflects the control conditions in reality. Therefore, it can be used as an alternative measurement index of control conditions and directly predict the possibility of behavioral incidence, with the veracity of the prediction dependent on the true extent of perceived behavioral control. At the same time, Ajzen (1991) believes that factors such as personal and social culture (such as experience, age, gender, social background, etc.) can influence the subject’s behavioral beliefs, indirectly influencing their behavioral attitude, subjective norm and perceived behavioral control, and finally influencing the subject’s behavioral intention and behavior. The Revised Model of Entrepreneurial Intention was proposed by Krueger and Carsrud (1993) to revise the entrepreneurial event model. The model holds that entrepreneurs’ entrepreneurial intention is mainly determined by three factors: entrepreneurial feasibility, entrepreneurial desire and entrepreneurial behavioral tendency.

As far as China’s theoretical research on social entrepreneurial intention is concerned, it is still in its infancy. Among Chinese scholars, Sheng (2008) was the first to regard social entrepreneurial intention as the key characteristic of the social entrepreneurial behavior subject in his research. Wang et al. (2014) divided the influencing factors of social entrepreneurship into subjective factors such as the comprehensive quality and ability of entrepreneurs themselves and objective factors such as entrepreneurship education and the entrepreneurship environment. Tang (2018) believes that there is an inverted “U” relationship between the social capital owned by social entrepreneurs and their social entrepreneurial intention, and entrepreneurs’ entrepreneurial self-efficacy plays a positive moderating role between the two. Generally speaking, Chinese scholars sorely lack research on social entrepreneurial intention, leaving an urgent need to conduct research on social entrepreneurial intention specifically for the Chinese market.

### Network Embeddedness, Sense of Opportunity Identification Efficacy and University Students’ Social Entrepreneurial Intention

#### Sense of Opportunity Identification Efficacy and University Students’ Social Entrepreneurial Intention

Entrepreneurship is a process of pooling a series of unique resources to pursue opportunities. Therefore, opportunity identification is the core concept of entrepreneurship research, whether in the field of commercial entrepreneurship or social entrepreneurship (Austin et al., 2012). Differing from commercial entrepreneurship, the distinguishing feature of social entrepreneurship opportunity identification is to solve social problems or create social values, which often focuses on serving basic and long-term needs more effectively through innovative ways (Peredo and Mclean, 2006). Shane and Venkataraman (2001) were the first to put forward the concept of the sense of opportunity identification efficacy. They define it from the perspective of opportunity source and believe that the sense of opportunity identification efficacy originates from the imbalance of an entrepreneur’s own knowledge and the asymmetry of access to information, and is the innovation demand caused by changes in environmental factors such as process, industry and population structure. Wood and Williams (2014) also point out from the perspective of internal mechanism that the sense of opportunity identification efficacy is the entrepreneur’s perception of opportunities through social rules and new organizations.

Some scholars have studied the relationship between the sense of opportunity identification efficacy and the entrepreneurial intention. Hill and Villa (1997) believe that the entrepreneurial process is extremely complicated, and quick and accurate identification of the opportunities over the process is a guarantee for entrepreneurs to implement actions, achieve goals and establish new businesses, which can directly affect the entrepreneurial intention of social entrepreneurs from a psychological point of view. Qing (2006) believes that when entrepreneurs recognize that there is a greater possibility of realizing success in entrepreneurship, or that there is a wider profit margin, they will generate more identity psychology, thus enhancing their motivation and confidence in the choice and enhancing their entrepreneurial intention. In other words, the higher the identification degree of entrepreneurial opportunities, the stronger the entrepreneurial intention and this leads to faster decision-making and more significant decision accuracy (Wei, 2014). Shepherd et al. (2015) also believe that identifying entrepreneurial opportunities promotes entrepreneurs’ judgment and controllability of the market, and can directly stimulate the growth of their entrepreneurial intention and the effectiveness of entrepreneurial decision-making. Thus, this paper believes that the sense of opportunity identification efficacy can have a direct effect on entrepreneurial intention to a certain degree.

However, university students differ from ordinary entrepreneurs. The disjunction between school and society makes them less sensitive to business information, and the efficiency of opportunity identification is vague. However, opportunity identification, as the prerequisite, core element and key link to entrepreneurship (Shane and Venkataraman, 2001), is an important factor that connects entrepreneurial intention and entrepreneurial behavior (Liu, 2018). Geroski (1995) points out that the ability of new enterprises to identify the environment, opportunities and cope with them by adjusting their own strategies will ultimately determine their chance of survival. And social entrepreneurship is also dissimilar from ordinary entrepreneurship, as its behavior is focused on solving social problems and realizing social values (Zahra et al., 2009; Béchervaise and Benjamin, 2013). Compared with traditional business entrepreneurs, social entrepreneurs have a stronger thirst for opportunities, policy support and access to resources (Chen, 2007). Therefore, it is of great significance to study the effect of the sense of opportunity identification efficacy on the social entrepreneurial intention of university students, a special group. This cannot only make up for the current theoretical gap, but also provide practical guidance for university students engaging in social entrepreneurship. Based on this, this paper puts forward the following assumptions:

Hypothesis 1: The sense of opportunity identification efficacy has a significant positive effect on the social entrepreneurial intention of university students.

#### Network Embeddedness and the Sense of Opportunity Identification Efficacy

Uzzi (1997) and Andersson et al. (2002) believed that network embeddedness is an important tool to study enterprises and is the basis for enterprises to connect and develop in economic activities and social networks. The concept of embeddedness was first put forward by Polanyi (1962), who believes that the human economic system and non-economic system are interrelated and influence each other, rather than function independently. Peng et al. (2019) believes that social entrepreneurial enterprises conform to different resources at different stages of their development, and that these resources can be divided into six areas: the enterprise’s material resources, technical resources, human resources, social networks, market resources and systems. In their latest research, Ma et al. (2019) provided good proof that an entrepreneurs’ network embeddedness has an influence on their sense of opportunity identification efficacy. Their research primarily focuses on entrepreneurs that had returned to China from overseas. This research proposed that these returning entrepreneurs’ network embeddedness was primarily overseas and, because of this, when they returned to start entrepreneurship, because they had weak network embeddedness in their new social network, they had a much poorer identification and application effect of human and social capital compared to local entrepreneurs, and this put them in a disadvantageous position.

Opportunity identification is the basic premise of entrepreneurship, and the resources and information needed in the process of opportunity identification are continuously transmitted and upgraded in the social network where the entrepreneur is located (Ozgen and Baron, 2007; Liu, 2014). Granovetter (1983) believes that diverse embeddedness structures of entrepreneurs in social relations will bring different information and knowledge, which will affect the deviation of entrepreneurs’ cognitive behavior. Subsequently, Burt and Burzynska (2017) in-depth study believes that entrepreneurs operating in structural holes in the social network structure generally have a better and faster opportunity identification ability and competitive advantage than entrepreneurs in other network locations, because the information and resources brought by different locations are diversified. Shane and Venkataraman (2001) also believe that the degree of diversification of entrepreneurs’ social network relationships is positively related to their ability to identify opportunities. Specifically, network relations with strong homogeneity can enable entrepreneurs to obtain more emotional support and practical help, realize more efficient information exchange and give them access to high-quality resources, thus improving the possibility of opportunity identification Burt and Burzynska (2017). While weak network relations formed by members of different industries can bring more heterogeneous resources to entrepreneurs, thus expanding the scope of entrepreneurial opportunities identified by entrepreneurs (Granovetter, 1983). Generally speaking, the more information entrepreneurs have, the stronger their tolerance of opportunity cost will be, and thus they have stronger entrepreneurial intention (Zhang, 2005). Lozano et al. (2016) believe that the social network structure of social entrepreneurs is one of the three entrepreneurial alertnesses, and its network quantity and diversity will help social entrepreneurs to find opportunities.

To summarize, the difference in network embeddedness directly affects the richness of information resources obtained by social entrepreneurs and their ability to identify entrepreneurial opportunities. The positive effect of network embeddedness on opportunity identification in the entrepreneurial process has been recognized by most scholars (Lin and Zhang, 2005; Ozgen and Baron, 2007). Therefore, this paper refers to Niu (2017) division of the network embeddedness of university students into two dimensions of network scale and network intensity, and puts forward the following assumptions:

Hypothesis 2a: Network scale has a significant positive impact on the sense of opportunity identification efficacy;
Hypothesis 2b: Network intensity has a significant positive effect on the sense of opportunity identification efficacy.

#### The Mediating Effect of the Sense of Opportunity Identification Efficacy on the Relationship Between Network Embeddedness and University Students’ Social Entrepreneurial Intention

Through the previous theoretical exposition, this paper has demonstrated that entrepreneurs’ network embeddedness has a significant impact on the sense of opportunity identification efficacy, and that the sense of opportunity identification efficacy also has a significant impact on social entrepreneurial intention. Referring to the mediating effect test method of Wang (2018), this paper boldly assumes that the sense of opportunity identification efficacy plays a mediating role in the interaction between network embeddedness and social entrepreneurial intention, but the specific full mediating role or partial mediating role needs to be further tested through empirical data. Based on this, this paper puts forward the following assumptions:

Hypothesis 3: The sense of opportunity identification efficacy plays a mediating role in the social entrepreneurial intention of university students in all dimensions of network embeddedness.

## Samples and Methods

### Samples

The empirical data in this paper come from investigations and interviews.

First of all, on the questionnaire design and sample of subjects. After sorting out the relevant literature, the author made a reference to the questionnaires of Wang (2014) and Niu (2017) of which reliability and validity have passed the tests. The questions on the questionnaire are presented in Tables 1–3. Because a pre-survey was first conducted in Sichuan, the province that the author is located in, it was found after analysis of the social entrepreneur data of students in Sichuan that over 60% of participants in the survey were studying business courses, therefore this sample limited the percentage of business course students to around 60%. Furthermore, of the academies of higher learning in Sichuan, Sichuan Normal University, University of Electronic Science and Technology of China, Southwest Jiaotong University and others were of a very high technical nature and could have an academic influence. Therefore, the sample in this paper was primarily taken from students from Sichuan University, which is a more comprehensive university, to alleviate the random academic influence.

**Table 1:** Measurement scale of university students’ social entrepreneurial intention.

Dimension	Item	Mean value	Factor loading	Credibility and validity index
University students’ social entrepreneurial intention	1. There is a very good chance that I will engage in social entrepreneurial activities within 5 years after I graduate.	2.74	0.8399	KMO Value = 0.899
	2. Sooner or later I will start my own business.	2.86	0.8630	
	3. I have already made specific plans to start my own business.	2.28	0.8585	Cronbanch’s α = 0.93
	4. I have already considered the industry and products I will work in the future.	2.80	0.8293	
	5. I have always been preparing to start my own business.	2.33	0.8935	Total variance contribution = 70.3%
	6. Even if I encounter practical difficulties, I will still choose to start my own business.	2.67	0.8666	
	7. I am willing to make time to study things related to social entrepreneurship.	3.41	0.7046	

**Table 2:** Measurement scale of network embeddedness.

Dimension	Item	Mean value	Factor loading	Credibility and validity index
Network scale	1. Compared to my classmates, I come into contact with and get to know more people.	3.25	0.8513	KMO value = 0.715
	2. Compared to my classmates, I come into contact with and get to know people in society on a greater scale.	3.21	0.8486	
	3. Compared to my classmates, I get a lot more support and help from people when I need it.	3.32	0.8497	Cronbanch’s α = 0.81
Network intensity	4. I keep frequent contact with people I know that I believe are important.	3.70	0.7869	
	5. Most of the people I know that I believe are important are relatives or friends.	3.94	0.7588	Total variance contribution = 63.75%
	6. I can always quickly get informative and effective help from others.	3.50	0.7550	
	7. The people I believe are important give me a lot of help concerning my career and my personal development.	3.68	0.7982	

**Table 3:** Measurement scale of sense of opportunity identification efficacy.

Dimension	Item	Mean value	Factor loading	Credibility and validity index
Sense of opportunity	1. I am good at identifying market segments.	3.11	0.8564	KMO value = 0.823
identification efficacy	2. I can identify valuable business opportunities.	3.11	0.9153	Cronbanch’s α = 0.898
	3. I can identify potential consumer or client requirements.	3.33	0.8714	
	4. I can develop new products or find ways to improve existing products.	3.10	0.8563	Total variance contribution = 76.59%

Second, as this paper’s questionnaire applies the Likert scale and all the items in the model are rated by the same groups of individuals, there is a possibility that measured variance appears in its results. Therefore, when conducting the questionnaire, the author considered Kar and George (2003) and randomly distributed the questionnaire items and ensured that subjects were isolated and times were staggered, to reduce the chance of subjects interfering with one another to the greatest degree and effectively reducing the chances of measured variance.

Last, a pre-survey was conducted in Sichuan University (the data was not used) and the questionnaire was revised according to the results to make it more accurate. In addition, we also set up an investigation team to interview the investigated university students face to face to ensure that they had a full cognition and understanding of each test item. The surveyed university students have voluntarily participated and we fully reserve the personal privacy and confidentiality of participants. Finally, 500 questionnaires were distributed and 483 were recovered in this survey, 466 of which were valid, with a total effective rate of 96.48%.

### Scale

All variables are measured on a 5-point Likert scale (1 for strongly disagree, 5 for strongly agree). In the 466 valid questionnaires, factor analysis and Cronbach’s Alpha coefficient test were conducted using software Stata 14, and 18 questions were aggregated into the 3 dimensions of university students’ social entrepreneurial intention, network embeddedness, and sense of opportunity identification efficacy. The following measures are taken, respectively.

#### University Students’ Social Entrepreneurial Intention

University students’ social entrepreneurial intention used as the dependent variable for this paper’s theoretical model. This paper uses the research of Chen et al. (1998), Han Lizheng (2009) and Kraus et al. (2012) as reference, and uses 7 items (shown in Table 1) to describe the characteristics of university students’ social entrepreneurial intention. The questionnaire data passes the reliability test (α = 0.93) and validity test (KMO = 0.899, Bartlett spherical test *p*-value < 0.01, factor load >0.5, cumulative variance rate = 70.3%).

This paper divides *the network embeddedness of university students* into two dimensions: network scale and network intensity (Niu, 2017). Network scale describes the number of members that entrepreneurs connect to in their current social network. It primarily uses the research and measurement methods of Wang (2011) for the design of 3 items. The network intensity indicates the closeness and intensity of the relationship between entrepreneurs and their connected members, which is mainly measured by referring to the research and measurement method of Granovetter (1983) to design the other 4 items and applies the 4 items, individual interaction rate, closeness of relationship, emotional intensity and reciprocal behavior to the 5-point Likert scale measurement. Finally, the 7 measure items of network embeddedness (shown in Table 2) all passed the reliability test (α = 0.81), and the cumulative variance rate of the two factors of network scale and network intensity is proposed to be 63.75% by the principal component analysis method. The analysis results are basically consistent with the expected model design.

*The sense of opportunity identification efficacy* is an intermediate variable. Referring to Chen et al. (1998), Han Lizheng (2009), this paper designed 4 items to collect data on the measurement scale of the sense of opportunity identification efficacy. The cumulative variance rate of α = 0.898 is 76.59%, KMO = 0.823, Bartlett sphere test *p*-value < 0.01, which indicates that reliability is within the acceptable range and the scale has a high level of validity.

#### Control Variables

This paper controls three variables, namely gender (Hechavarría et al., 2017; Lortie et al., 2017), educational level (Hörisch et al., 2016; Estrin et al., 2016) and professional background (Fan and Wang, 2005), which may have a great influence on the social entrepreneurial intention of college students and the sense of opportunity identification efficacy of intermediary variables. Descriptive statistics on these three control variables are shown in Table 4. Estrin et al. (2013) believe that women have a stronger concept of caring than men, so they are more inclined to the social entrepreneurial behavior of creating social value than commercial entrepreneurship (Hechavarría et al., 2017). Different scholars hold varied views on the influence of educational level on social entrepreneurial intention. According to the empirical results of Estrin et al. (2016), the higher the educational level, the more likely it is to choose social entrepreneurship; However, Hörisch et al. (2016) believe that the improvement of education level will lead entrepreneurs to prefer mature enterprises and weaken their social entrepreneurial intention.

**Table 4:** Descriptive statistics of samples.

Items	Class	Number of samples	Percentage (%)
Gender	Male	102	21.9
	Female	364	78.1
Education background	College	14	3
	Undergraduate	446	95.7
	Master	5	1.1
	MBA	1	0.2
Specialty	Literature, history, and philosophy	12	2.6
	Economics	116	24.9
	Management	292	62.7
	Law	4	0.9
	Pedagogy	11	2.3
	Natural science	14	3
	Engineering	13	2.8
	Agronomy	3	0.6
	Medical and military	1	0.2

### Methods

This paper used the verification of sense of opportunity identification efficacy as an intermediary variable to reveal the mechanism of action that network embeddedness has on a university student’s social entrepreneurial intention. The various variable definitions can be seen in Table 5. There were many methods of analyzing and testing intermediary effect. This paper, in line with Wen and Ye (2014) research, takes the existing independent variable NE, intermediary variable SOIE, 3 control variables and the dependent variable USSEI as its model to consider NE passing SOIE and its influence on USSEI using the following test procedures:

**Table 5:** Definition of variables.

	Variables	Definition
Dependent variable	University students’ social entrepreneurial intention	USSEI, referring to Chen et al. (1998), Han Lizheng (2009), Kraus et al. (2012), described using the 7 items in Table 1
Independent variable	Network embeddedness	NE, divided into network scale and network intensity. With network scale referring to Wang (2011) and described in the 3 items in Table 2; and network intensity referring to Granovetter (1983) and described in the 4 items in Table 2
Mediating variable	Sense of opportunity identification efficacy	SOIE, referring to Chen et al. (1998), Han Lizheng (2009) and described in the 4 items in Table 3
Control variable	Gender	1 male; 0 females
	Education background	EB, primarily divided into college level and below and undergraduate, masters, Ph.D. and above
	Specialty	SPE, students of business related course restricted to around 60%

First, regression analysis of dependent variable USSEI to independent variable NE. At this point the independent variable NE’s coefficient should reach a significant level, or it would cease intermediary effect analysis; as in equation 1:

$$USSEI\;=\;\alpha \;+\;\beta_{0}NE\;+\;\beta_{1}Gender\;+\;\beta_{2}EB\;+\;\beta_{3}Spe\;+\;\varepsilon \;\;\;\;\;\;\;\;\;\;\mathrm{(1)}$$

Second, intermediary variable SOIE to independent variable NE regression; as in equation 2, assuming that NE’s coefficient is β_0_ at this point.

$$SOIE\;=\;\alpha \;+\;\beta_{0}NE\;+\;\beta_{1}Gender\;+\;\beta_{2}EB\;+\;\beta_{3}Spe\;+\;\varepsilon \;\;\;\;\;\;\;\;\;\;\mathrm{(2)}$$

Third, dependent variable USSIE with independent variable NE and intermediary variable SOIE regression; as in equation 3, assuming that NE’s coefficient is β_4_ and SOIE’s coefficient is β_5_.

$$USSEI\;=\;\alpha \;+\;\beta_{4}NE\;+\;\beta_{5}SOIE\;+\;\beta_{6}Gender\;+\;\beta_{7}EB\;+\;\beta_{8}Spe\;+\;\varepsilon \;\;\;\;\;\;\;\;\;\;\mathrm{(3)}$$

Finally, the successive coefficient testing of β_0_ andβ_5_. If β_0_ and β_5_ are both significant then the testing coefficient isβ_4_. If it is significant, then there is the existence of a partial intermediary effect and if it is not significant, then there is the existence of a complete intermediary effect. These are the traditional steps for conducting testing on regression coefficient; if at least one of β_0_ or β_5_ is not significant, then Sobel testing will be conducted. If this test passes, then intermediary effect exists and if it does not pass, then it proves that the intermediary effect does not exist.

## Statistical Results and Analysis

### Descriptive Statistics and Relevant Analysis

Before regression analysis, the variance expansion factor VIF is used to exclude the multicollinearity test of the model (each VIF < 1.6). The correlation coefficient analysis of each variable is shown in Table 6. The results show that there is a significant positive correlation between the sense of opportunity identification efficacy and the social entrepreneurial intention of university students (*r* = 0.6390^∗^), and the two dimensions of network embeddedness (network scale and network intensity) are a significantly positive correlation with the sense of opportunity identification efficacy (r_network scale_ = 0.5020^∗^, r_network intensity_ = 0.4227^∗^), which preliminarily verifies the assumptions H1, H2a, and H2b mentioned above.

**Table 6:** Correlation coefficient of variables.

	Entrepreneurial intention	Network scale	Network intensity	Sense of opportunity identification efficacy	Gender	Education background	Specialty
Entrepreneurial intention	1.0000						
Network scale	0.4635*	1.0000					
Network intensity	0.2898*	0.5087*	1.0000				
Sense of opportunity identification efficacy	0.6390*	0.5020*	0.4227*	1.0000			
Gender	0.1818*	0.0480	-0.0662	0.1381*	1.0000		
Education background	-0.0566	-0.0163	-0.0217	0.0450	-0.0578	1.0000	
Specialty	-0.0065	-0.0717	-0.0736	-0.0614	0.0865	0.0001	1.0000

^∗^*p < 0.1*.

### Regression Analysis

#### Sense of Opportunity Identification Efficacy and University Students’ Social Entrepreneurial Intention

In this study, multiple linear regressions are used to verify the relationship between the sense of opportunity identification efficacy and university students’ social entrepreneurial intention. The data results are shown in Table 7. The explained variables of the two models are university students’ social entrepreneurial intention. The explanatory variables of Model 1 only include control variables to verify the influence of individual gender, educational level and professional background on their entrepreneurial intention; Model 2 adds explanatory variables on the basis of control variables to the sense of opportunity identification efficacy. The results show that after adding explanatory variables, the revised R^2^ increases from 0.029 to 0.419, with a significant positive correlation between the sense of opportunity identification efficacy and university students’ social entrepreneurial intention (β = 0.777^∗∗∗^), which indicates that the higher the sense of opportunity identification efficacy of entrepreneurs, the stronger the social entrepreneurial intention, assuming H1 is satisfied. The reason for this result may be that opportunity identification helps entrepreneurs to master the market and make timely controllable adjustments, thus generating positive feedback on their entrepreneurial psychology, which is consistent with the research results of Shepherd et al. (2015).

**Table 7:** Sense of opportunity identification efficacy and university students’ social entrepreneurial intention.

Variables	Model 1	Model 2
Gender	0.422***	0.204**
	(3.606)	(2.310)
Education background	-0.200	-0.347***
	(-1.014)	(-2.709)
Specialty	-0.018	0.020
	(-0.465)	(0.699)
Sense of opportunity identification efficacy		0.777***
		(17.017)
Constants	3.086***	0.854***
	(7.462)	(2.928)
Number	466	466
R^2^	0.036	0.424
Adjustment of R^2^	0.029	0.419

^∗∗^*p < 0.05, ^∗∗∗^*p* < 0.01; Robust regression is used for regression*.

#### Network Embeddedness and Sense of Opportunity Identification Efficacy

In order to explore the relationship between network embeddedness and sense of opportunity identification efficacy, two models were designed in this paper. The regression results are shown in Table 8. The explained variables of the two models are the sense of opportunity identification efficacy. Model 1 only adds control variables as independent variables; Model 2 adds two dimensions to explain the network embeddedness of variables, network intensity and network scale. The regression results show that after adding network embeddedness, the revised R^2^ of the model is increased from 0.021 to 0.306, and the coefficients of network intensity and network scale are significantly positive (β_network scale_ = 0.327^∗∗∗^, β_network intensity_ = 0.267^∗∗∗^), which indicates that the stronger the network intensity and the larger the network scale, the higher the entrepreneur’s sense of opportunity identification efficiency, and the above assumptions H2a and H2b are satisfied. Network scale and network intensity represent to some extent the convenience and reliability of entrepreneurs in obtaining information and resources, and efficient information exchange and resource acquisition can enhance entrepreneurs’ identification and grasp of opportunities (Krueger and Day, 2009; Burt and Burzynska, 2017).

**Table 8:** Network embeddedness and sense of opportunity identification efficacy.

Variables	Model 1	Model 2
Gender	0.280***	0.271***
	(2.919)	(3.364)
Education background	0.189	0.228
	(0.721)	(1.609)
Specialty	-0.049	-0.019
	(-1.351)	(-0.483)
Network scale		0.327***
		(6.914)
Network intensity		0.267***
		(3.818)
Constants	2.873***	0.626
	(5.456)	(1.627)
Number	466	466
R^2^	0.027	0.313
Adjustment of R^2^	0.021	0.306

^∗∗∗^*p < 0.01; Robust regression is used for regression*.

#### The Mediating Effect of the Sense of Opportunity Identification Efficacy Between Network Embeddedness and University Students’ Social Entrepreneurial Intention

This paper chooses multiple linear regressions to study the relationship among sense of opportunity identification efficacy, network embeddedness and university students’ social entrepreneurial intention, and the mediating effect of sense of opportunity identification efficacy on network embeddedness and university students’ social entrepreneurial intention. The three models all take university students’ social entrepreneurial intention as dependent variables, respectively, introduces control variables, network embeddedness and sense of opportunity identification efficacy as independent variables, and gradually carries out hierarchical regression analysis. Through Table 9, it is found that the coefficient of network scale in Model 2 is significantly positive (β = 0.443^∗∗∗^), while the coefficient of network intensity is not significant (β = 0.126), which indicates that university students’ social entrepreneurial intention is positively related to their network scale and has nothing to do with network intensity. Therefore, the mediating effect of sense of opportunity identification efficacy on network intensity and entrepreneurial intention is no longer considered. Through the observation of Model 3, it is found that after adding the variable of the sense of opportunity identification efficacy, the correction R^2^ increased from 0.248 to 0.453, and the coefficient of the sense of opportunity recognition efficacy is significantly positive (β = 0.670^∗∗∗^), indicating that the mediating effect of the sense of opportunity identification efficacy is significant. The powerful connection between network scale and entrepreneurial intention from Model 2 to Model 3 decreases (β decreases from 0.443 to 0.224), which indicates that the relationship between network scale and entrepreneurial intention has changed clearly after the intermediary variable sense of opportunity identification efficacy is added. This also verifies that the influence of network scale on university students’ social entrepreneurial intention is realized by influencing their sense of opportunity identification efficacy, i.e., the sense of opportunity identification efficacy plays a part in mediating the relationship between network embeddedness and university students’ social entrepreneurial intention, assuming H3 is partially satisfied.

**Table 9:** Mediating effect of sense of opportunity identification efficacy on network embeddedness and university students’ social entrepreneurial intention.

Variables	Model 1	Model 2	Model 3
Gender	0.422***	0.384***	0.203**
	(3.606)	(3.821)	(2.351)
Education background	-0.200	-0.167	-0.320**
	(-1.014)	(-1.189)	(-2.374)
Specialty	-0.018	0.013	0.026
	(-0.465)	(0.314)	(0.902)
Network scale		0.443***	0.224***
		(7.852)	(4.099)
Network intensity		0.126	-0.053
		(1.649)	(-0.865)
Sense of opportunity identification efficacy			0.670***
			(11.824)
Constants	3.086***	1.011**	0.592*
	(7.462)	(2.428)	(1.852)
Number	466	466	466
R^2^	0.036	0.248	0.453
Adjustment of R^2^	0.029	0.240	0.445

^∗^*p < 0.1, ^∗∗^*p* < 0.05, ^∗∗∗^*p* < 0.01; Robust regression is used for regression*.

## Discussion

Based on the research of existing scholars on the sense of opportunity identification efficacy (Shane and Venkataraman, 2001, etc.), network embeddedness (Granovetter, 1983; Uzzi, 1997, etc.) and social entrepreneurial intention (Dwivedi and Weerawardena, 2018, etc.), this paper discusses the action mechanism among the three by designing a multiple linear regression model. The main conclusions are as follows: (1) Sense of opportunity identification efficacy can significantly stimulate university students’ social entrepreneurial intention. Because opportunity identification helps entrepreneurs to grasp the market and make timely controllable adjustments, thus generating positive feedback on their entrepreneurial psychology (Shepherd et al., 2015); (2) The network embeddedness (network scale and network intensity) of entrepreneurs is also significantly positively correlated with their sense of opportunity recognition efficacy. Because the larger the scale and intensity of the network where the entrepreneur is located, the deeper and broader information can be brought to the entrepreneur, thus facilitating their opportunity identification and grasp; (3) Through the hierarchical regression model, it is found that the sense of opportunity identification efficacy can only partially mediate the relationship between network embeddedness and university students’ social entrepreneurial intention, which is mainly manifested in the positive correlation between university students’ social entrepreneurial intention and their network scale, and has nothing to do with network intensity.

It is worth noting that when conducting multiple linear regression and hierarchical regression analysis, we found that after adding the explanatory variable, the sense of opportunity identification efficacy on the basis of control variables, the impact of academic qualifications on college students’ social entrepreneurial intention becomes significant, and the higher the academic qualifications, the smaller their social entrepreneurial intentions, which is consistent with the research results of Forbes (2005) and Hörisch et al. (2016), because when the academic qualifications of individual entrepreneurs reach a certain level, they are more inclined to manage mature enterprises with more choices. However, the effect of educational background on the sense of opportunity identification efficacy has not changed much before and after joining the network embeddedness. In terms of gender, its influence on the sense of opportunity identification efficacy and the social entrepreneurial intention of university students has remained significant. However, since this paper has not conducted a more detailed study on gender, this can become the next direction of research. In terms of university students’ majors, their influence on the sense of opportunity identification efficacy and the social entrepreneurial intention of university students has not changed much and is not significant, which is consistent with the research results of Chinese scholars (Fan and Wang, 2005), but it may also be due to the fact that the proportion of students majoring in economics and management in this survey is as high as 87.6%. Therefore, the next step is to compare different professional levels of the interviewed university students in groups.

Generally speaking, Chinese university students can enhance their sense of opportunity identification efficiency by expanding the scale and intensity of their social network, realize a timely grasp of various opportunities and chances in their entrepreneurial process, and use this psychological factor to maximize their entrepreneurial intention and form good positive feedback on their social entrepreneurial process.

## Theoretical Contributions

This paper primarily builds a theoretical model of “network embeddedness-sense of opportunity identification efficacy-university students’ social entrepreneurial intention.” Compared with previous case studies, this paper primarily explains the action mechanism among the three from an empirical perspective through a multivariate linear model. The theoretical significance of this paper is primarily summarized and listed in the following points:

First, this paper supplements research into entrepreneurial intention in the realm of social entrepreneurship, particularly as a focused study on university students. Due to the short rise of social entrepreneurship and the lack of a mature system for overall development, its theoretical research needs to be improved. At present, research on social entrepreneurship focuses more on the concept definition of social entrepreneurship related topics and the sorting out of the existing research (Du Jingjing, 2015; Fu et al., 2017). However, in-depth research on university students’ social entrepreneurial intention definitely adds color to the theoretical research in the field of social entrepreneurship.

Second, this paper expands research on the relationship between network embeddedness and social entrepreneurial intention. At present, the theoretical and empirical research on entrepreneurial intention is mainly in the field of commercial entrepreneurship (Simsek et al., 2015). Research on entrepreneurial intention in the realm of social entrepreneurship is relatively lacking, while research into the relationship between entrepreneurs and their network embeddedness is even more lacking. This paper uses network embeddedness as its independent variable and social entrepreneurial intention as its dependent variable, to not only research the correlation between the two, but also import the sense of opportunity identification efficacy, this meditating variable, to display the mechanism of action between the two. And in turn, provides a good supplement into research on the relationship between network embeddedness and social entrepreneurial intention.

Third, promote the discipline integration of social cognitive theory and social entrepreneurial research. This paper innovatively imports the concept of the “sense of opportunity identification efficacy” as a mediating variable conducting research on the individual psychological level of social entrepreneurs faced with the network embeddedness and social entrepreneurial intention as a mechanism of action, and discovered that the network scale of university students can have a significant influence on their sense of opportunity identification efficacy, which increases their timely grasp on various opportunities during the process of entrepreneurship.

## Practical Significance

The model of “network embeddedness-sense of opportunity identification efficacy-university students’ social entrepreneurial intention” constructed in this paper has not only specific theoretical value, but also considerable practical significance.

First, entrepreneurial intention is the guiding indicator of entrepreneurial behavior. Theoretical research on entrepreneurial intention can be converted into leading guidance for entrepreneurial behavior and is a prerequisite for entrepreneurship. Positive entrepreneurial intention can increase the incubation rate of social entrepreneurial behavior, thus, to a certain extent, making up for the deficiencies of government failure, market failure and charity failure, and contributing more strength to China’s poverty eradication, employment solution and improvement of the social environment.

Second, a sense of opportunity identification efficacy, as an essential factor for social entrepreneurs, can represent entrepreneurs’ behavior orientation and dynamics to a large extent. The research in this paper will be helpful to, from the social entrepreneurs themselves, fundamentally help entrepreneurs carry out the reasonable adjustment of daily social entrepreneurial activities, and give full play to their subjective initiative and strengthen their social entrepreneurial intention.

Third, as one of the main forces of entrepreneurship in China, this paper shows the influencing factors of social entrepreneurial intention in students that have a certain guiding significance in maximizing their social entrepreneurial behavior. By expanding the sense of opportunity identification efficacy of university students and network embeddedness through reasonable methods, they can enhance their social entrepreneurial intention and promote the achievement and realization of their social mission.

## Limitations and Prospects

### Limitations

A limitation in the subjects of this questionnaire exists. This questionnaire adopted an offline survey method, mainly covering students from several universities in Chengdu, and due to time and geographical limitations, it does not have a certain randomness and is relatively limited.

There is no follow-up survey on entrepreneurial intention. Entrepreneurial intention, as the subjective attitude of entrepreneurs, is an element of dynamic change, which is not followed up in this study.

### Prospects

Due to the limitations of current data, later research can use the spread and extensiveness of the Internet to expand the scope of and increase the number of samples and adopt more representative sample data for research. However, this also requires researchers to strengthen the ability of information identification, screening and analysis. In addition, this paper also mentions that there has not been any further detailed research on the gender and professional level of the interviewed university students, which will become the direction of research in the next stage.

## Ethics Statement

An ethics approval was not required as per applicable institutional and national guidelines and regulations. The informed consent of the participants was implied through survey completion.

## Author Contributions

YT conceptualized and designed the empirical research and wrote this manuscript. WW took charge in designing the empirical research, collecting data, and wrote this manuscript. YL designed the empirical research and performed the data analysis. TZ, JL, and HL performed the data analysis, and revised the manuscript. All authors worked collectively and significantly contributed to this manuscript.

## Conflict of Interest Statement

The authors declare that the research was conducted in the absence of any commercial or financial relationships that could be construed as a potential conflict of interest.
